# Editorial: (Re)defining hypothyroidism: the key to patient-centered treatment

**DOI:** 10.3389/fendo.2024.1369723

**Published:** 2024-02-06

**Authors:** Antonio C. Bianco, Colin M. Dayan, Jacqueline Jonklaas

**Affiliations:** ^1^ Section of Adult and Pediatric Endocrinology, Diabetes and Metabolism, University of Chicago Medicine, Chicago, IL, United States; ^2^ Thyroid Research Group, Division of Infection and Immunity, Cardiff University, Cardiff, United Kingdom; ^3^ Division of Endocrinology and Metabolism, Georgetown University Medical Center, Washington, DC, United States

**Keywords:** thyroid, hypothyroidism, levothyroxine, liothyronine, quality of life

Hypothyroidism was first described in the 19^th^ century, with effective treatment introduced approximately two decades later. Despite large experiences accumulated over a century, some modern-day patients continue to experience persistent symptoms even while appropriately treated from a biochemical standpoint.

While a stated goal of the treatment of hypothyroidism is to resolve symptoms, most physicians focus on the normalization of serum TSH values, which is the marker used to diagnose hypothyroidism and adjust the replacement dose of levothyroxine. However, complete physiological replacement has not been possible in other hormone deficiency syndromes, and it may be presumptuous to assume that it can easily be achieved in hypothyroidism. Given the residual symptoms and metabolic abnormalities experienced by some patients, there is therefore a need to question and redefine therapeutic success in hypothyroidism. Potential issues to consider are whether there are other biomarkers of thyroid status, in addition to TSH, that may be important, and whether different biomarkers are important for different tissues. In addition, the relevance of the etiology of the hypothyroidism and how to balance benefits in long-term versus immediate clinical outcomes should be considered. Perhaps endpoints also need to be more rigorously compared with endogenous euthyroidism.

This Research Topic seeks to compile research that (re)defines success in the treatment of patients with hypothyroidism beyond the normalization of TSH levels. It contains fundamental aspects of thyroid hormone physiology and action (Salas-Lucia and Bianco), as well as an extensive discussion of questions around the diagnosis and treatment of hypothyroidism with special emphasis on patient care. The discussion starts with the acknowledgment that many thyroid- and non-thyroid-related factors affect the patient response to treatment of hypothyroidism, in terms of their satisfaction with therapy. In particular, the etiology of hypothyroidism, the amount of residual thyroid function that the patient retains, and the presence of thyroid peroxidase antibodies. In her article, Jonklaas discusses recent studies addressing the ultimate significance of these factors and their effect on determining patient-reported outcomes, quality of life, and patient satisfaction.

It is clear that, given the chronic nature of hypothyroidism and non-specific characteristics of the symptoms, defining the nature of the symptoms that remain once biochemical euthyroidism has been achieved is of critical importance. This is not an easy task, though. Jansen et al. discuss the difficulties around attributing symptoms to their underlying cause, and the need for physicians to acknowledge and be willing to manage symptoms without a clear pathological correlate (3). Along these lines, Cramon et al. provide a road map for how the utilization of patient-reported outcomes (PROs) can enhance communication with patients and improve patient satisfaction, symptom management, and quality of life. In their article, they provide an overview of thyroid-related PROs, describe unsolved quality-of-life issues, provide examples of routine collection of PROs, and summarize key points facilitating the successful implementation of thyroid-related PROs in routine clinical practice.

The effectiveness of treatment of hypothyroidism with levothyroxine at doses that normalize serum TSH levels is also discussed. Although this has become the standard of care worldwide, randomized, prospective clinical trials that assess alternative approaches are scarce. The potential relevance of monitoring serum T3 levels during treatment with levothyroxine is extensively discussed (Salas-Lucia and Bianco). In addition, Fitzgerald and Falhammar question the wisdom of using serum TSH to adjust the dose of levothyroxine altogether. There is evidence suggesting that treatment of hypothyroxinemia, regardless of the TSH level, and monitoring therapy using FT4 and/or triiodothyronine levels, depending on the replacement regime, may result in more successful treatment of hypothyroidism than relying on TSH levels for patient selection and subsequent treatment monitoring.

Furthermore, Heald et al. discuss the emerging evidence that may account for the efficacy of liothyronine, either in combination with levothyroxine or as desiccated thyroid extract (contains a mixture of T4 and T3), in people who are symptomatically unresponsive to levothyroxine. Although randomized controlled trials (RCT), have not established greater efficacy for the LT3 + LT4 combination than for LT4 alone, it is conceivable that the trial designs may not have been optimal, with the use of unphysiological, short-acting LT3 preparations and non-thyroid-specific patient-reported outcome measures contributing as well. This is extensively discussed by Premawardhana et al., who also make a series of recommendations for the design of future RCTs of LT3 + LT4 compared to LT4 alone.

Lastly, Ettleson and Papaleontiou provide a critical analysis of the health outcomes literature in patients with treated hypothyroidism. To date, overall mortality, cardiovascular morbidity and mortality, bone health, and cognitive function have been evaluated as endpoints in clinical outcomes studies in patients with treated hypothyroidism. From a healthcare quality perspective, the treatment of hypothyroidism should be evaluated not just on its effectiveness for the individual patients but also to the extent to which patients of different sociodemographic groups are treated equally.

After having considered the treatment of hypothyroidism as a resolved issue, it is fascinating to see how the medical community has realized that much remains to be discovered and improvements are badly needed in this area. This realization has sparked the interest of pharmaceutical companies and has led funding agencies to support basic investigation as well as numerous clinical trials. These are exciting times for the thyroid community and for the patients who suffer from hypothyroidism. We hope this Research Topic will contribute to the improvement of their care.

## Author contributions

AB: Writing – original draft, Writing – review & editing. CD: Writing – original draft, Writing – review & editing. JJ: Writing – original draft, Writing – review & editing.

